# Assessing the accuracy of forest above-ground biomass and carbon storage estimation by meta-analysis based close-range remote sensing

**DOI:** 10.48130/forres-0025-0017

**Published:** 2025-08-21

**Authors:** Jincheng Liu, Zhuo Chen, Ziyu Zhao

**Affiliations:** 1 College of Natural Resources and Environment, Northwest A& FUniversity, Yangling 712100, Shaanxi, China; 2 State Key Laboratory of Information Engineering in Surveying, Mapping and Remote Sensing, Wuhan University, Wuhan 430079, Hubei, China; 3 Department of Resource Management, Tangshan Normal University, Tangshan 063000, Hebei, China

**Keywords:** Forest, Remote sensing, Point cloud, Carbon storage, Biomass, Meta-analysis

## Abstract

The swift progress of close-range remote sensing necessitates a quantitative evaluation of its accuracy in estimating forest above-ground biomass (AGB) across diverse scales, forest types, methodologies, and variables. These evaluations will enhance the effectiveness of remote sensing in forest monitoring, reveal the carbon sequestration capability of forest vegetation, and underscore the critical function of forests as terrestrial carbon sinks. In this study, we designated *R*^2^ as the effect size for the meta-analysis given that it is commonly regarded as a measure for estimating accuracy in AGB research, which indicates the explanatory capacity of independent variables. Utilizing 187 global investigations and 233 datasets, this research systematically assessed the accuracy (*R*^2^) of ground light detection and ranging (LiDAR), unmanned aerial vehicles (UAVs), spectra, and red–green–blue (RGB) sensors across the single-tree, plot, and stand scales. The discrepancies in accuracy across the various research methods and the independent variables in the allometric growth equation were also assessed. The research indicated that ground lidar exhibited the best accuracy across all studies and was the most effective approach at both the single-tree and plot scales. Nonetheless, as the scale of the research broadened, both accuracy and sample size diminished. Furthermore, the variations from different approaches among different forest types were substantial; therefore, it was necessary to model these forest types explicitly. By integrating diameter at breast height (DBH or D) and tree height (H) as independent variables in the allometric growth equation, the method showed improved estimation accuracy. The estimation of AGB must address the issue of accumulated error arising from the interconversion of DBH and H, single-tree segmentation, and specific allometric growth equations, which are subsequently compounded at the plot and stand levels. Close-range remote sensing is currently the most efficient method for estimating forest AGB, surpassing conventional measurement techniques. Yet, due to sensor limitations, no single sensor achieved optimal results independently. The integration of multi-source data and scale adaptation strategies further enhanced the efficacy of close-range remote sensing, surpassing the conventional survey methods. Moving forward, efforts should prioritize cross-platform data standardization, deep learning model refinement, and the establishment of non-destructive validation systems to support high-precision forest carbon monitoring, in alignment with carbon management goals.

## Introduction

Forests sequester atmospheric CO_2_ in trees, understory vegetation, and soil through photosynthetic processes^[1−3]^, contributing approximately 76%–98% of the planet's terrestrial organic carbon pool^[4−6]^. This vast carbon reservoir serves as a cornerstone in mitigating climate change and curbing the greenhouse effect ^[7]^. The development of high-resolution mapping of forest above-ground biomass (AGB), which underpins the assessment of forests' carbon sequestration capacity, has become integral to national climate strategies and the broader pursuit of green, low-carbon development. Accordingly, the scientific and precise estimation of forest AGB and carbon storage has emerged as a focal and extensively contested topic within the research community.

Forest AGB serves as an important indication for analyzing forests' community structure and ecosystem integrity^[8]^, as well as a key measure for evaluating the composition and quality of forest ecosystems^[9]^. The assessment of forest AGB has evolved from traditional field-based inventories to remote sensing-driven methodologies^[10]^, leading to an integrated "satellite–airborne–terrestrial" research paradigm^[11]^. In recent years, substantial advances in close-range remote sensing technologies^[12]^—including unmanned aerial vehicles (UAVs), ground-based platforms, and portable sensing instruments—have greatly increased their relevance in forest vegetation monitoring. Among them, UAV-based AGB estimation has emerged as a key method for assessing forest carbon sinks^[13,14]^. This monitoring system allows for fine-scale delineation of near-ground structural features, effectively resolving the limits of classic standard-tree assessment techniques in heterogeneous stands with varying growth patterns and ages. Furthermore, it reduces observer-induced errors and labor intensity^[15]^, solidifying its position as a reliable and efficient tool for current AGB evaluation. Close-range remote sensing methods fall into four categories: red–green–blue (RGB)-based^[16]^, spectrum-based^[17]^, light detection and ranging (lidar)-based^[18−20]^, and radar-based^[21,22]^. While these methodologies enable multi-scale observations ranging from single trees to entire plots and stands, a pronounced trade-off between spatial scale and estimation accuracy persists across different platforms. For example, ground lidar systems offer sub-centimeter precision in retrieving structural parameters at the single-tree level^[23,24]^, yet their accuracy tends to diminish when scaled up to plot-level analyses due to cumulative errors in single-tree segmentation. Conversely, UAV-based lidar excels in large-scale AGB assessments by delivering reliable estimates of tree height (H) and canopy structure, though it is constrained by limited canopy penetration and reduced efficacy in single-tree segmentation^[25−27]^. Notably, AGB estimation is also modulated by factors such as the species composition, stand structure, and forest age^[28]^. Given that most natural forests comprise mixed-species assemblages rather than homogeneous monocultures, the application of species-specific AGB models and carbon conversion coefficients becomes imperative. Accordingly, there is a pressing need to examine the determinants of estimation accuracy across multiple spatial scales in tandem with prevailing research. These accuracy-related variables not only vary across observational scales but are also intricately linked to forest typologies, model inputs, and methodological frameworks—dimensions whose influence on AGB estimation remains insufficiently quantified.

Forest AGB estimation methods using close-range remote sensing can be broadly categorized into two methodological frameworks: The first integrates ground plot data with allometric equations^[29,30]^, while the second combines a limited number of ground plots with remote sensing data, which can be further subdivided into parametric and non-parametric approaches^[31,32]^. These methodologies facilitate AGB estimation across multiple spatial scales, from single trees to plots, and ultimately to regional or landscape levels. The allometric approach, incorporating field-measured parameters into species-specific or region-specific equations, is widely regarded as the most accurate technique for single-tree AGB estimation. This method mitigates the challenges posed by labor-intensive and destructive sampling, while simultaneously enhancing the precision of AGB estimates at both the plot and stand scales^[33]^, thereby establishing itself as the prevailing strategy for forest AGB assessments^[34]^. At the plot level, the integration of high-precision individual tree segmentation with localized allometric models further enables robust and spatially explicit AGB estimation. However, the scalability of this approach to broader regions is constrained by cumulative errors and model generalization limitations. To address these challenges, parametric and non-parametric methods leverage the outputs of allometric models as the ground truth to interpolate discrete tree-level measurements into continuous plot-scale AGB surfaces, thereby minimizing the fieldwork demands and enabling extrapolation to regional scales^[35−38]^. Parametric models typically rely on statistically significant correlations between vegetation indices and AGB to construct predictive equations, allowing rapid estimates of AGB across large areas. In contrast, non-parametric approaches employ machine learning or deep learning algorithms in conjunction with close-range remote sensing data, effectively overcoming the limitations of parametric techniques, particularly under conditions involving high-dimensional variables or complex, non-linear relationships between the spectral features and AGB. These emerging methodologies offer promising avenues for future research, facilitating rapid, cost-effective, and non-destructive multi-scale estimates of forest AGB^[36,39,40]^.

Against the backdrop of global climate imperatives, achieving the twin goals of peak carbon peak and carbon neutrality (i.e., the carbon management goals in China) has become a pressing policy priority^[41]^, thereby underscoring the urgency of advancing methodologies for estimating forest carbon stocks and enabling the rapid, accurate assessment of forest carbon storage through close-range remote sensing^[42]^. In this context, a comprehensive meta-analysis grounded in extensive empirical data is essential to elucidate the accuracy and reliability of close-range remote sensing techniques across varying spatial scales.

This study aims to address the following scientific questions: (1) How can we use meta-analyses to quantify the effects of scale, forest type, methodology, and independent variables on the accuracy of AGB estimations? (2) How can we enhance estimates of forest AGB through the integration of multi-source remote sensing data? (3) What are the future directions for the development of close-range remote sensing monitoring systems?

## Materials and methods

### Literature search and screening

Relevant literature evaluating the stability of multi-scale forest AGB estimation based on close-range remote sensing was retrieved from the China National Knowledge Infrastructure database (www.cnki.net), the Wanfang Database (wanfangdata.com.cn), Google Scholar (https://scholar.google.com), VIP Database (https://cqvip.com), and Web of Science (www.webofscience.com). The search employed a set of keywords including 'optical remote sensing', 'UAV lidar', 'ground lidar', 'above-ground biomass', 'point cloud', 'multi-source remote sensing data', 'photogrammetry', and 'forest'. The literature retrieval period spanned from 1 January 2010 to 15 May 2025. In total, over 2,000 English-language publications and more than 400 Chinese-language studies were identified.

To ensure the accuracy and reliability of this study, the retrieved literature was screened in two stages on the basis of strict inclusion criteria. The initial screening involved reviewing the titles and abstracts to eliminate studies with irrelevant research areas, themes, methodologies, subjects, or data types. Duplicates and review articles were also excluded. Subsequently, a second round of screening was conducted according to the following criteria. First, studies had to be based on field experiments, excluding those conducted in greenhouse settings or involving potted plants. Second, eligible studies were required to report the standard deviation (SD), standard error (SE), and sample size (*n*) of the relevant variables, as well as the coefficient of determination (*R*^2^) for the estimation results. Studies using alternative evaluation indices in place of *R*^2^ were excluded. Mean values and standard deviations were obtained either directly from tables or extracted from figures using the Get Data Graph Digitizer software (http://getdata-graph-digitizer.com). Third, only studies focusing on AGB were included. Research involving belowground biomass, soil carbon storage, deadwood carbon, or litter carbon was excluded. Fourth, studies were limited to forest ecosystems, with those focused on grasslands, deserts, or other biomes excluded. Fifth, only literature utilizing close-range remote sensing platforms was retained, while studies relying solely on airborne or satellite remote sensing were excluded. Lastly, when extracting *R*^2^ values, only the highest reported value for a given model was selected. In cases involving different spatial scales, tree species, or experimental sites, each was treated as an independent observation.

**Figure 1:**
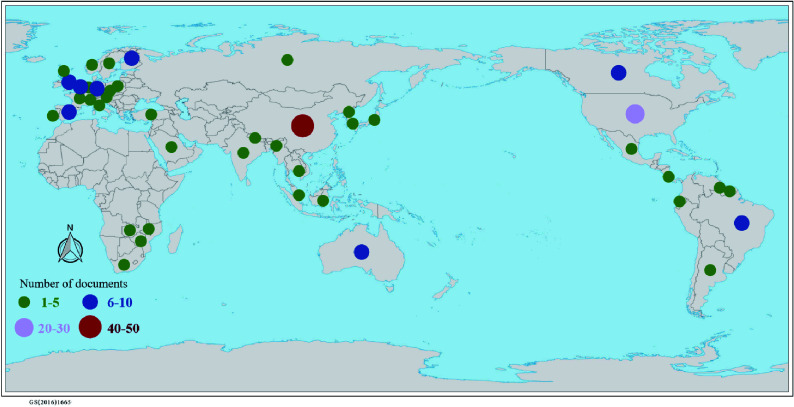
Distribution of global literature.

A total of 187 articles and 233 research data were collected, which were widely distributed and representative (Fig. 1). The database contained more than 1,000 variables, including the first author, year, sample size, SE, and other data (Supplementary Table S1).

### Data calculation and processing

**Figure 2:**
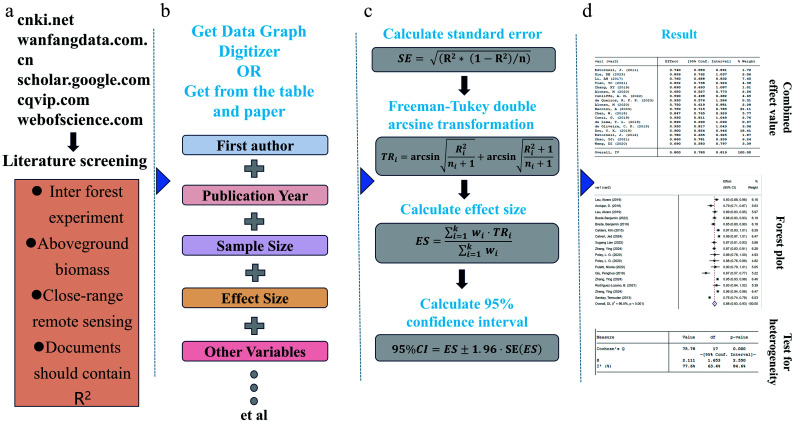
Workflow of the meta-analysis. (a) Literature screening and search process; (b) extracting relevant data from the literature; (c) calculating the ES and 95% confidence interval (CI); (d) result.

The present study adopted a single-group rate meta-analysis (Fig. 2) to quantitatively synthesize the *R*^2^ reported in individual studies evaluating the accuracy of forest AGB estimations. Unlike traditional meta-analyses that compare effect sizes (ESs) between intervention and control groups, this approach consolidates single-group summary metrics (*R*^2^) to generate a pooled estimate of the model's goodness-of-fit across heterogeneous experimental contexts. As *R*^2^ is widely recognized as a robust indicator of estimation accuracy in AGB modeling, representing the proportion of variance in the dependent variable explained by the independent variables, it was designated as the ES for this analysis. In single-rate meta-analysis, accurate calculation of the standard error is critical for capturing the uncertainty inherent in each study's ES. Accordingly, the standard error corresponding to each individual *R*^2^ value was computed and employed to weight the relative contribution of each study in the overall aggregation. The standard error for single-group rates was calculated using the following formula Eq. (1):

1 $ {S E}_{i}=\sqrt{({R}_{i}^{2}\cdot (1-{R}_{i}^{2})/{n}_{i})} $

where, $ {S E}_{i} $, $ {R}_{i}^{2} $, and $ {n}_{i} $ are the standard deviation, *R*^2^, and sample size of the *i-*th sample, respectively.

To overcome the inherent statistical limitation of variance being intrinsically linked to the mean, a variance-stabilizing transformation was applied prior to data synthesis. Specifically, the Freeman–Tukey double arcsine transformation Eq. (2) was utilized to normalize the distribution of *R*^2^ values and to alleviate the effects of heteroscedasticity^[43]^ $ {TR}_{i} $ (transformed $ {R}_{i} $) stabilizes the variance of coefficients of determination across studies through double arcsine transformation. This adjustment is critical for pooling accuracy estimates in random-effects meta-analysis.

2 $ {TR}_{i}=\mathrm{a}\mathrm{r}\mathrm{c}\mathrm{s}\mathrm{i}\mathrm{n}\sqrt{\dfrac{{R}_{i}^{2}}{{n}_{i}+1}}+\mathrm{a}\mathrm{r}\mathrm{c}\mathrm{s}\mathrm{i}\mathrm{n}\sqrt{\dfrac{{R}_{i}^{2}+1}{{n}_{i}+1}} $

It is important to note that when the sample sizes are small and the ES values are close to 1, the confidence interval of the ES may exceed the theoretical bounds of [0, 1] following arcsine transformation. This phenomenon results from variance inflation during the inverse transformation process and represents a statistical artifact arising from the temporary expansion of the value domain in the course of meta-analytic aggregation, rather than a reflection of statistical unreliability. All such estimates were corrected using Freeman–Tukey adjustment to ensure conformity with the theoretical limits.

The pooled ES denotes the synthesized estimate of the overall rate, obtained by statistically integrating the findings from multiple independent studies. It serves as the most reliable approximation of the true probability of the target outcome within the broader population, while accounting for inter-study variability and heterogeneity. The 95% confidence interval (95% CI) is a critical indicator of the estimate's precision, signifying that there is a 95% likelihood that the true ES resides within this interval. The transformed pooled ES was computed using the inverse variance weighting method Eq. (3), and the corresponding 95% CI was subsequently derived Eq. (4).

3 $ ES=\dfrac{\sum _{i=1}^{k} {w}_{i}\cdot {TR}_{i}}{\sum _{i=1}^{k} {w}_{i}} $

4 $ 95{\%}CI=ES\pm 1.96\cdot \mathrm{S}\mathrm{E}\left(ES\right) $

where Var represents variance, $ {w}_{i}=\dfrac{1}{\mathrm{V}\mathrm{a}\mathrm{r}\left({TR}_{i}\right)} $, and SE represents the standard error.

In the meta-analysis, heterogeneity among the included studies was evaluated using the χ^2^ test and the *I*^2^ statistic. The χ^2^ test produces a *p*-value to assess the statistical significance of heterogeneity, while the *I*^2^ statistic quantifies the proportion of total variation attributable to true heterogeneity rather than random error. A low *p*-value coupled with a high *I*^2^ value indicates substantial heterogeneity across studies, whereas a high *p*-value and low *I*^2^ value suggest relative homogeneity. To compute the *p*-value, Cochran's *Q* statistic was first calculated Eq. (5), which quantifies the dispersion in the accuracy of AGB estimation among studies. Under the null hypothesis of homogeneity, *Q* follows a χ^2^ distribution with *k* – 1 degrees of freedom, where *k* represents the number of included studies. The χ^2^ is generated by the software, and the *p*-value is determined by comparing *Q* with the corresponding χ^2^ distribution Eq. (6). Subsequently, the *I*^2^ statistic was derived to assess the magnitude of heterogeneity, based on the relationship between *Q* and its degrees of freedom Eq. (7).

5 $ Q=\sum _{i=1}^{k} {w}_{i}(E{S}_{i}-\overline{ES}{)}^{2} $

6 $ P=P\left({\chi }_{df}^{2} > Q\right) $

7 $ {I}^{2}=\left[\right(Q-(k-1))/Q{]} \cdot 100{\%} $

where $ E{S}_{i} $ is the ES of the *i*-th study, *k* is the number of studies, and *df* is the degree of freedom *df* = k – 1.

Values of *p* ≥ 0.10 or *I*^2^ ≤ 50 % indicated that there was no heterogeneity in the data and that the fixed effect model would be used for the combined analysis. Otherwise, *p* ≤ 0.10 or *I*^2^ ≥ 50 % indicated that there was statistical heterogeneity among the studies and that the random effect model then would be used for combined analysis. From the test results, only RGB at the stand scale and mixed forest, UAV lidar at the shrub scale, and ground lidar at the coniferous forest had poor heterogeneity, and the fixed effect model was used. All subgroups at other scales used the random effect model.

All relevant data were compiled and organized using Microsoft Excel 2021 (Microsoft, USA). The pooled ES and 95% CI values were computed using Stata 15SE (StataCorp LLC, USA), and forest plots were generated with PyCharm 2023 (JetBrains, Czech Republic).

### Data packet

**Figure 3:**
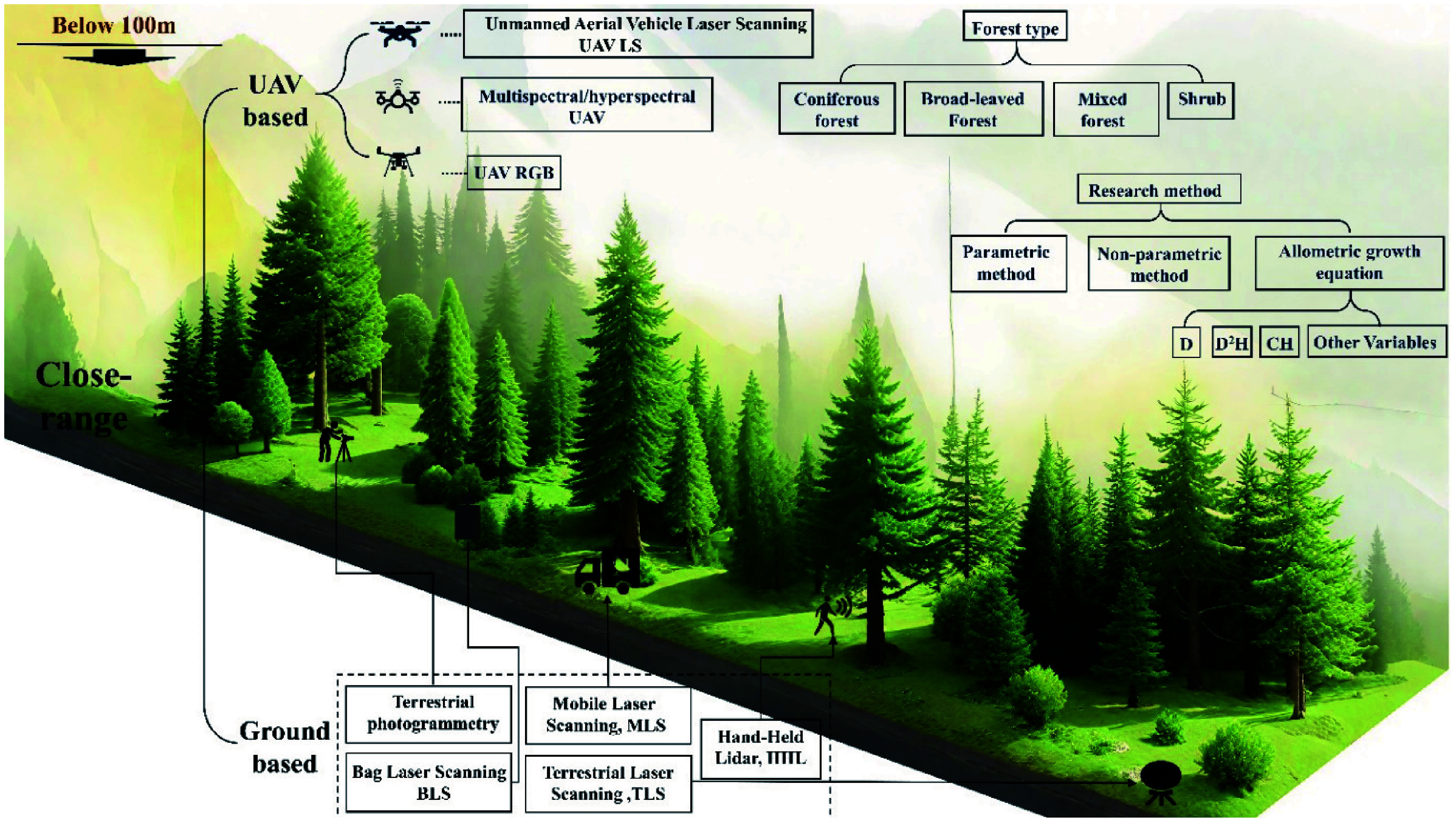
AGB estimation systems using close-range remote sensing.

In this study, "close-range" remote sensing refers to non-contact measurement systems operating at altitudes less than or equal to 100 m above ground level. This classification included both ground-based and UAV-based platforms. To facilitate the analysis, sensors were categorized into three types according to their sensor modality: RGB sensors, spectrum sensors, and lidar sensors (Fig. 3). lidar sensors were further classified according to the platform height into ground lidar and UAV lidar. Ground lidar encompassed terrestrial laser scanning (TLS), mobile laser scanning (MLS), backpack laser scanning (BLS), and hand-held lidar (HHL). In contrast, RGB and spectrum sensors were not classified by platform height; for example, UAV-based photogrammetry and ground-based photogrammetry were collectively categorized as RGB sensors. Currently, most methods for estimating AGB using radar rely on satellite data, with limited research utilizing UAV-based radar systems. Therefore, radar-based approaches were not included in the scope of this study. However, with the increasing availability and application of UAV-based radar technology, future studies are expected to integrate it into close-range remote sensing frameworks for AGB estimation.

This study further examined the impact of forest type on the accuracy of AGB estimation by classifying forests into four structural categories: coniferous forest, broadleaf forest, mixed forest, and shrubs. This classification framework enabled a systematic evaluation of close-range remote sensing performance across distinct vegetation structure. AGB estimation approaches were subsequently grouped into two primary categories: remote sensing synergy methods integrated with ground plots and allometric equations, and remote sensing synergy methods combined with a limited number of ground plots. The latter category was further subdivided into parametric and non-parametric models to assess their respective impacts on estimation accuracy.

Within the allometric growth equation framework, independent variables were classified according to combinations of diameter at breast height (DBH or D), crown width (CW), height (H), and other variables. The final classifications comprised D^2^H, H, D, and other variables (including unconventional combinations such as crown volume and crown density). This classification enabled a detailed analysis of how different independent variables affected estimation accuracy.

## Results

### Accuracy at different research scales

**Figure 4:**
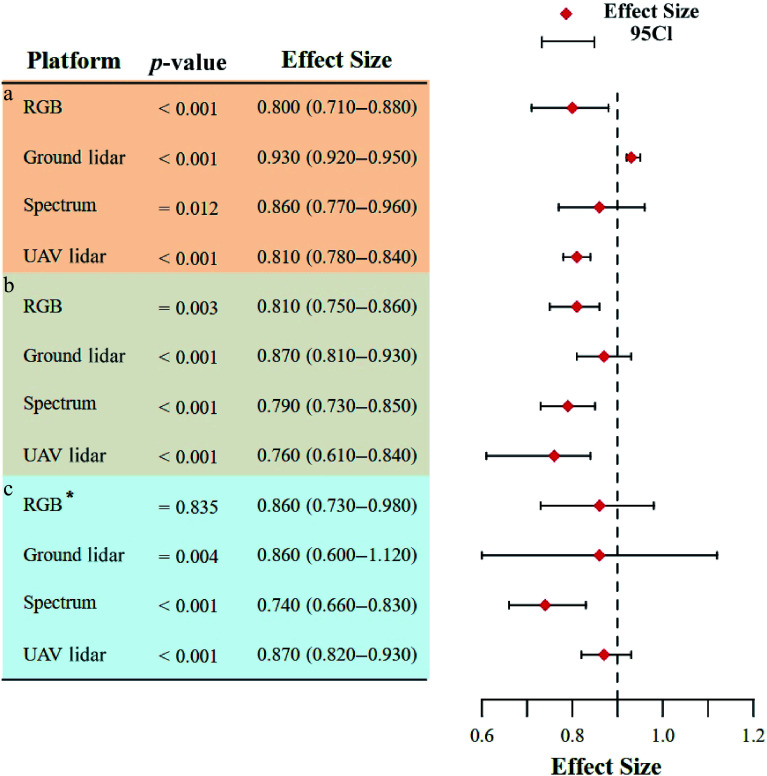
Effects of different research scales on forest AGB estimation: (a) Single-tree scale; (b) plot scale; (c) stand scale. * indicates that a fixed effects model was used.

The meta-analysis revealed that the research scale significantly influenced the accuracy of close-range remote sensing techniques (Fig. 4). At the single-tree scale (which accounted for 56.55% of the sample size, Table 1), ground lidar exhibited the highest pooled ES (ES = 0.93; 95% CI: 0.92–0.95), significantly outperforming UAV lidar (ES = 0.81; 95% CI: 0.78–0.84) and RGB (ES = 0.8; 95% CI: 0.71–0.88). This discrepancy arose from the subcentimeter point cloud resolution of ground lidar^[23]^, which allowed the direct extraction of DBH and H, facilitating high-precision fitting of the D^2^H allometric growth equation. In contrast, UAV lidar and RGB were constrained by limited canopy penetration, which led to missing subcanopy structures and higher segmentation errors, prevented direct acquisition of single-tree diameter parameters, and ultimately reduced the accuracy of estimation at this scale.

**Table 1:** Statistical table of sample size at different research scales.

Scale of study	Sensor type	Number of samples	Sample proportion (%)	*I*^2^ (%)
Tree	UAV lidar	38	19.90	97.6
	Ground lidar	42	21.99	75.8
	RGB	24	12.57	95.8
	Spectra	4	2.09	72.7
Plot	UAV lidar	31	16.23	97.0
	Ground lidar	7	3.66	73.5
	RGB	13	6.81	59.5
	Spectra	12	6.28	68.3
Stand	UAV lidar	13	6.81	86.6
	Ground lidar	2	1.05	88.3
	RGB	3	1.57	0.0
	Spectra	2	1.05	90.8
Total		191	100	

At the plot scale (which accounted for 32.98% of the sample size), the ground lidar also achieved the highest pooled ES (ES = 0.87; 95% CI: 0.81–0.93), suggesting its reliability in AGB estimation at this scale. However, UAV lidar exhibited an unexpectedly low ES (ES = 0.76; 95% CI: 0.61–0.84) compared with its theoretical capacity for rapid large-scale acquisition, attributed to point cloud sparsity and computational limitations in data processing^[44]^.

At the stand scale (which accounted for 10.48% of the sample size), all methods except spectra (ES = 0.74; 95% CI: 0.66–0.83) achieved high ES, which was sufficient to prove their reliability at this scale. Although the ground lidar had the highest ES on all three scales, the increase in the research scale reduced the sample size and the accuracy, so the workload and uncertainty were significantly improved^[45]^. Therefore, the use of UAV RGB, especially UAV lidar, should be the preferred method at the stand scale^[46]^.

### Accuracy in different forest types

**Figure 5:**
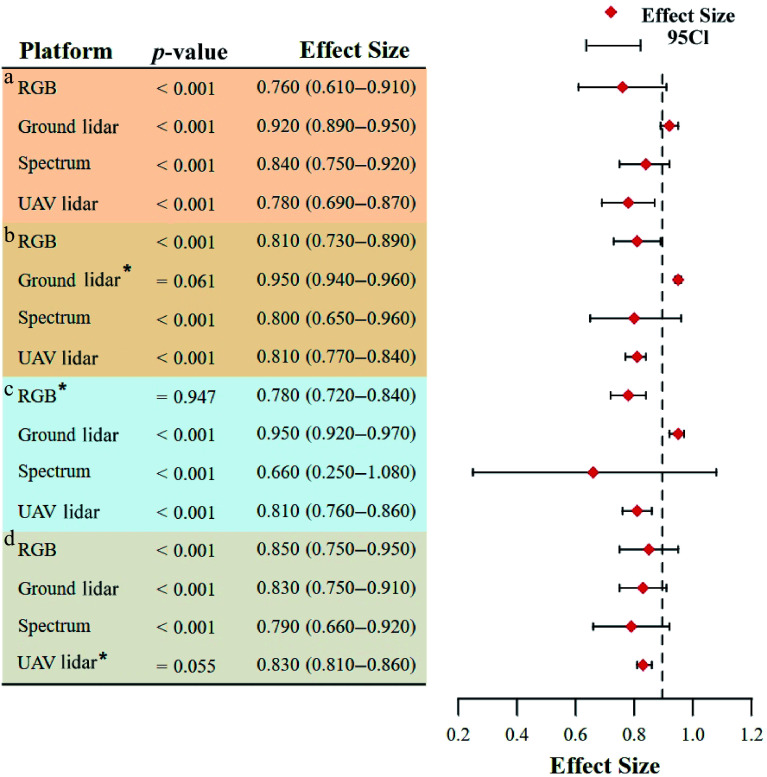
Effects of different forest types on forest AGB estimations: (a) broadleaf forests; (b) coniferous forests; (c) mixed forests; (d) shrubs. * indicates that a fixed effects model was used.

There were significant differences in technical performance among different forest types (Fig. 5). Ground lidar had the highest ES in all forest types. In addition, the spectra (ES = 0.84; 95% CI: 0.75–0.92) had the highest ES in broadleaf forests (which accounted for 20.43% of the sample size, Table 2). In coniferous forests, RGB had higher accuracy (ES = 0.81; 95% CI: 0.73–0.89) than the other three sensor types. It may be that the texture features of RGB images were easier to extract due to the single-canopy structure. In the mixed forest, the spectra (ES = 0.66; 95% CI: 0.25–1.07) were limited by the signal saturation of dense canopy. The accuracy of AGB estimation in shrubs was generally low (RGB ES = 0.85; 95% CI: 0.75–0.95; ground lidar ES = 0.83; 95% CI: 0.75–0.91). Because the vertical structure of low vegetation was difficult to capture by lidar, it was necessary to combine high-resolution ground RGB images to extract the data on plant density^[47]^.

**Table 2:** Statistical table of samples in different forest types.

Type of forest	Sensor type	Number of samples	Sample proportion (%)	*I*^2^ (%)
Broadleaf forests	UAV lidar	22	11.52	98.2
	Ground lidar	14	7.33	42.1
	RGB	11	5.76	97.7
	Spectra	6	3.14	83.3
Coniferous forests	UAV lidar	20	10.47	85.8
	Ground lidar	12	6.28	42.1
	RGB	11	5.76	77.4
	Spectra	4	2.09	83.3
Mixed forests	UAV lidar	31	16.23	97.7
	Ground lidar	17	8.90	80.9
	RGB	6	3.14	0.0
	Spectra	3	1.57	99.6
Shrubs	UAV lidar	9	4.71	47.4
	Ground lidar	8	4.19	80.6
	RGB	12	6.28	88.8
	Spectra	5	2.62	86.0
Total		191	100	

### Accuracy with different research methods

**Figure 6:**
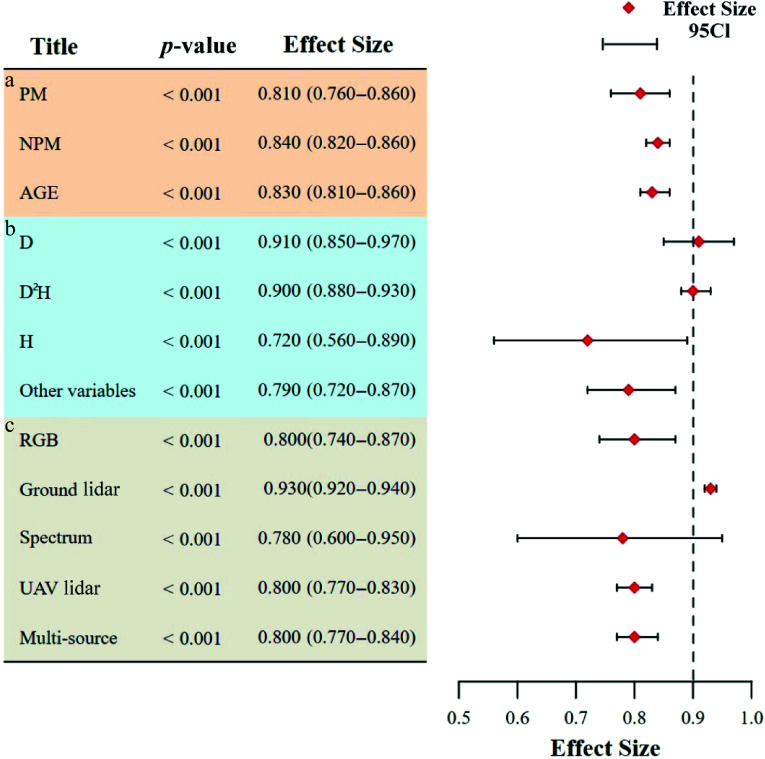
AGB estimation accuracy of different research methods, different independent variables, and different platforms. (a) Different research methods. PM, parametric method; NPM, non-parametric method, AGE, allometric growth equation. (b) Different independent variables. (c) Accuracy comparison between multi-source remote sensing and individual remote sensing data sources.

The results showed that (Table 3, Fig. 6a) there was no significant difference in accuracy among the three methods (*p* < 0.001). As the standard method of traditional AGB estimation, the allometric growth equation obtained the highest combined ES (ES = 0.83; 95% CI: 0.81−0.86). The non-parametric method (ES = 0.82; 95% CI: 0.80−0.85) also achieved good performance which was basically the same as that of the allometric growth equation (ΔES = 0.01), probably due to the complex non-linear relationship between remote sensing features and AGB that machine learning algorithms can capture. In addition, the non-parametric method had high flexibility and was more adaptable to forests with multiple species and complex structures^[48]^. In contrast, the parametric method showed lower accuracy (ES = 0.80; 95% CI: 0.76−0.85), which might be limited by the problem of signal saturation in dense canopies where the vegetation index was insensitive.

**Table 3:** Statistical table of samples with different research methods.

Research method	Number of samples	Sample proportion (%)	*I* ^2^
Parametric method	95	40.77	98.6
Non-parametric method	62	26.61	91.9
Allometric growth equation	76	32.62	97.2
Total	233	100	

### Accuracy with different independent variables

The results (Table 4; Fig. 6b) showed that D^2^H (ES = 0.90; 95% CI: 0.88−0.93) and D (ES = 0.91; 95% CI: 0.85−0.97) had the highest ES. Therefore, the allometric growth equation with D as the independent variable had high accuracy in estimating AGB. D is a commonly used variable in forest surveys. Due to their mature measurement methods and high accuracy, they provide reliable basic data for AGB estimations. In contrast, models with only H as an independent variable had the lowest effect (ES = 0.72; 95% CI: 0.56−0.89). This difference might arise from inaccuracies in measuring H and the fact that H may not adequately reflect trees' morphological characteristics nor fully capture all factors influencing AGB^[29]^. Conversely, other variables (ES = 0.79; 95% CI: 0.72–0.87), such as those derived from crown height equations or equations using crown volume and crown density as predictors, offer advantages. These independent variables are readily obtainable, as they can be directly retrieved from UAV lidar data. This facilitates large-scale rapid estimation and mapping of AGB, capabilities that are often lacking when using traditional allometric equations.

**Table 4:** Statistical table of sample with different independent variables.

Independent variable	Number of samples	Sample proportion	*I* ^2^
H	14	18.42%	98.4
D	10	13.16%	77.4
D^2^H	28	37.04%	93.5
Other	24	31.58%	95.3
Total	76	100%	

### Multi-source vs. single-sensor accuracy

Multi-source data collaboration has been a hot spot in AGB estimation over recent years, as well as in close-range remote sensing research. Therefore, this study compared the accuracy of multi-source close-range remote sensing data with that of single-sensor remote sensing data (Fig. 6c). Looking at the results alone, it appeared that multi-source data (ES = 0.8; 95% CI: 0.77−0.84, Table 5) did not significantly improve the estimation accuracy of AGB compared with single data sources and were even lower than the ES of ground lidar (ES = 0.93; 95% CI: 0.92−0.94). However, from the perspective of specific research contexts, two key factors contributed to this outcome. First, substantial heterogeneity existed across forest plots. Multi-source remote sensing was typically applied to forests characterized by complex vertical stratification and dense horizontal structures—environments that were often inaccessible to manual survey efforts. In such cases, relying on a single data source, particularly ground lidar, was unlikely to yield comparable accuracy^[49]^. Second, differences in the research scale played a critical role. Ground lidar was predominantly employed at the single-tree and plot scales, whereas multi-source remote sensing was more frequently utilized for large-scale AGB estimation^[34]^. At such broader scales, the accuracy of estimation tended to decline, and the inherent complexity of forest structure further exacerbated this reduction in precision^[50,51]^. Therefore, the coordinated integration of multi-source data emerged as a robust and effective strategy, enabling compensation for the limitations of individual data types and facilitating more accurate estimations of AGB under structurally complex stand conditions.

**Table 5:** Statistical table of samples using different sensors.

Sensor	Number of samples	Sample proportion	*I* ^2^
UAV lidar	82	35.19%	97.3
Ground lidar	52	22.32%	75
RGB	40	17.17%	93.5
Spectra	18	7.73%	99.7
Multi-source	41	17.60%	95.5
Total	233	100%	

## Discussion

### Accuracy at single-tree scale

The meta-analysis revealed that the research scale played a decisive role in shaping the estimation accuracy of close-range remote sensing technologies. At the single-tree scale, ground lidar exhibited significantly higher ES than UAV lidar, underscoring its superior performance in this context. This advantage stemmed from the capabilities of both TLS and mobile laser scanning (MLS), which excelled in capturing the three-dimensional structural attributes of individual trees with high precision^[23,52−54]^. These systems demonstrated remarkable accuracy in estimating total biomass, stem biomass, and branch biomass, and numerous studies have confirmed that ground lidar has reached a mature level of application for single-tree AGB estimation, with predictive accuracy approaching unity. In contrast, UAV lidar-based estimation at the single-tree scale typically relied on generating a canopy height model (CHM) by subtracting a digital elevation model (DEM) from a digital surface model (DSM) derived from point cloud data ^[24]^. This process requires extensive supplementary spatial information, such as ground control points and high-resolution digital terrain models ^[32]^. Alternatively, single-tree segmentation algorithms were employed to extract structural parameters, such as DBH, H, and C, which were then incorporated into allometric equations for estimating AGB^[29,55]^. As a result, the precision of DBH and H extraction has become critical to the reliability of estimation. However, UAV lidar faces inherent limitations at low operating altitudes, including canopy occlusion and laser pulse attenuation or scattering, which hinder effective penetration into subcanopy layers^[56]^. This constraint significantly compromised the acquisition of key understory information, thereby reducing the accuracy of single-tree AGB estimates. In recent years, to address these limitations, researchers have sought to capitalize on UAV lidar's strengths in capturing canopy-level metrics such as H and CW^[30,57,58]^. These features could be used to infer DBH^[19]^ or to apply allometric models that do not require DBH as an input, offering alternative pathways for accurate AGB estimation in structurally complex forest environments.

Furthermore, the H and CW parameters extracted from UAV lidar could be effectively integrated with DBH measurements derived from ground lidar to enhance the accuracy of AGB estimation^[25,59]^. In addition, several studies have demonstrated that the fusion of UAV lidar with hyperspectral imagery markedly improved the explanatory power of AGB estimation models^[60]^, thereby enabling high-resolution mapping of forest AGB^[48,61]^. These findings collectively indicated that for high-precision AGB estimation at the single-tree scale, ground lidar should be regarded as the preferred method. Nevertheless, its application remains constrained by substantial time investment and limited spatial coverage, presenting practical challenges for large-area implementation.

### Accuracy at plot and stand scales

When the scale was extended to the plot scale, the ground lidar and RGB methods showed high accuracy. Ground lidar is unable to measure areas that are difficult to reach, whereas UAV-based lidar is beneficial for conducting high-resolution tree canopy mapping in steep mountainous areas. It complements traditional methods and has thus been widely used for estimating forest AGB. Nevertheless, the measurement accuracy of UAV lidar at the small plot scale has not yet been widely recognized, as the point cloud data are relatively sparse^[62,63]^. The main reason for this result was that it could not overcome the problem of error accumulation in the process of single-tree estimation because of its limitation at the single-tree scale. This error not only exists in the calculation of single-tree AGB, but also in single-tree segmentation^[64]^. Compared with the allometric growth equation, the non-parametric method has obvious advantages in estimating AGB on a large spatial scale. The non-parametric method could avoid the accumulated error in the estimations of single trees by using a small amount of plot data as samples, and a more robust and accurate estimation model could be developed through continuous training, which could be applied to estimating the AGB of sample plots and large areas^[37,39]^. These methods improved the estimation accuracy at the plot scale to a certain extent, but it was still significantly lower than that of ground lidar. Therefore, when estimating AGB at the single-tree and plot scales, ground lidar should be used as the preferred scheme. However, it should be noted that while ground lidar could provide detailed three-dimensional structural parameters of individual trees, without high-precision individual tree segmentation, accumulated errors from scale extrapolation (e.g., from single-tree to plot level) would significantly degrade the final accuracy.

In contrast, the performance of spectral methods at the stand scale remained suboptimal. This limitation primarily arose from the susceptibility of spectral data to atmospheric interference, such as cloud and fog cover, which significantly compromised the accuracy of data acquisition^[65,66]^. Furthermore, the inherent complexity of the forest's vertical structure often resulted in the attenuation or loss of critical spectral signals^[67,68]^, thereby impeding optimal performance. As a consequence, the applicability of spectral approaches in large-scale, humid tropical rainforests was markedly constrained. Additionally, data saturation was a common issue in high-biomass environments^[69]^. In regions characterized by dense vegetation cover or elevated leaf area index, the saturation of vegetation indices—particularly those relying on near-infrared reflectance—was especially pronounced^[68]^. This saturation effect led to systematic underestimation of high biomass values, diminishing the overall accuracy of vegetation index-based AGB estimations. The complexity of forest ecosystems further complicated the relationship between vegetation indices and biomass. For instance, in structurally heterogeneous environments, the correlation between the near-infrared band and biomass was substantially weaker compared with more homogeneous conditions^[70]^. Moreover, many forest AGB models are based on stand-level structural variables, such as DBH and H^[71]^, parameters that are often unavailable in annually updated forest inventory datasets. This mismatch contributed to pronounced discrepancies between modeled estimates and ground truth observations. Collectively, these factors underscored the considerable limitations of relying solely on spectral methods for estimating forest AGB at the stand scale.

As the scale of research broadens, sensor selection must increasingly prioritize the comprehensiveness and representativeness of the acquired data to counteract the adverse effects of error accumulation on the accuracy of estimation. An expanding body of literature has underscored the value of integrating multi-source data across spatial scales and sensing platforms to enhance both the precision and reliability of forest AGB assessments. For example, ground lidar was frequently utilized to extract key structural parameters such as DBH and H, which were subsequently employed to construct AGB estimation models that are applicable to large-scale forested landscapes^[72,73]^. This methodology is often complemented by auxiliary datasets—such as UAV lidar, hyperspectral, and multi-spectral imagery—for model support and validation of the accuracy. Such multi-source fusion not only significantly improves the precision of estimation but also effectively reduces the labor intensity and logistical complexity of large-scale data acquisition ^[26,74]^.

### Accuracy in different forest types

The complexity of forest types exerted a profound influence on the accuracy of AGB estimation, primarily through variations in spectral characteristics and canopy structure ^[65,75]^. Coniferous forests, characterized by their regular canopy architecture, were well-suited to the application of stereo registration algorithms in RGB imagery^[76,77]^. In broadleaf forests, the biochemical and morphological uniformity of canopy foliage contributed to stable, linear relationships between spectral vegetation indices and AGB, facilitating accurate estimations^[47,78]^. In contrast, mixed forests presented considerable structural complexity, with heterogeneous horizontal canopy arrangements and intricate vertical layering. These features induced multiple scattering of the spectral signals within the canopy, leading to signal saturation and a marked reduction in the accuracy of spectral estimation (ES = 0.66 in mixed forests, compared with ES = 0.84 in broadleaf forests and ES = 0.80 in coniferous forests). Furthermore, vegetation indices often failed to adequately suppress background interference^[79]^, particularly from exposed soil, thereby introducing additional noise and reducing the models' precision^[80]^.

Compared with models that relied exclusively on spectral data, integrated approaches that combined spectral information with structural data—such as lidar or photogrammetric metrics—consistently yielded higher accuracy in estimating forest AGB across diverse forest types^[10,56]^. The findings highlighted the pronounced variation in estimation performance across both methodological approaches and forest types. At present, no universally applicable model exists for large-scale multi-species AGB estimation. Therefore, distinguishing forest types during model development is essential for enhancing the accuracy of estimation and ensuring robust application across heterogeneous forested landscapes ^[81]^.

### Accuracy with different research methods

The convergence in accuracy between ground-plot methods employing allometric equations and non-parametric approaches underscored the scale-dependent nature of methodological selection. At the single-tree level, the reliability of allometric models was predominantly determined by the precision of input variables and the inherent robustness of the equations themselves; higher fidelity in these parameters directly enhanced the accuracy of AGB estimation^[50]^. Measurement errors might arise from instrumental limitations, necessitating repeated sampling for correction, or from procedural inaccuracies. Moreover, AGB estimates derived through allometric models—whether targeting individual branches, foliage, or entire stands—were susceptible to the propagation of cumulative error, which can significantly distort AGB estimates at the plot or landscape scale. Such error transmission also occurred when DBH was inferred from H or vice versa^[82]^. Increasing the sampling frequency and implementing standardized measurement protocols have proven effective in mitigating the propagation of error^[83,84]^. It was also shown that model-related errors often stem from the geographically constrained applicability of allometric equations, which are typically region-specific and lack universal generalizability. Parametric methods, which established relationships between remote sensing-derived metrics and forest AGB, faced similar constraints in parameter accuracy, frequently caused by mismatches in spatial resolution or inconsistencies in measurement techniques^[82,85]^. Regrettably, extracting robust predictive variables from raw datasets remains a challenge, particularly when data integrity is compromised by factors such as cloud cover disrupting the acquisition of the normalized differenced vegetation index or suboptimal atmospheric correction degrading the accuracy of surface reflectance.

Non-parametric approaches, supported by machine learning and deep learning techniques, offer a compelling solution to the limitations of parametric models by effectively capturing complex, non-linear relationships between remote sensing variables and AGB. Among these, deep learning exhibits considerable promise for estimating forest AGB, enabling the extraction of nuanced features related to vegetation density, species composition, and carbon storage, thus presenting a robust alternative to conventional regression-based frameworks. Nevertheless, the application of deep learning within close-range remote sensing contexts remains relatively underexplored and warrants greater scholarly attention. Recent studies in forestry have demonstrated the efficacy of deep learning models in predicting key structural parameters such as H and CW^[38,86]^.

Accordingly, future research should prioritize the adaptation and refinement of established deep learning architectures to better accommodate the heterogeneous conditions of forest ecosystems across different biogeographical regions and forest types^[87]^. Advancements in this area will not only foster the integration of innovative computational methodologies in forest science but also provide critical technical support for the precise quantification of forest carbon stocks.

### Accuracy with different independent variables

The results of the meta-analysis showed that D and D^2^H had the highest accuracy, and H had the lowest accuracy. The ES of D was slightly higher than that of D^2^H, indicating that its accuracy was more reliable. However, the accuracy of the D^2^H composite variable fluctuated less (95% CI: 0.81–0.93), indicating that it was more stable in most cases. The primary error associated with D^2^H stemmed from inaccuracy in estimating H. Although some studies have suggested that H is a powerful indicator for AGB estimation, accurate estimation of H has remained a significant challenge in forestry research, regardless of the method employed^[88]^. Additionally, other studies have shown that incorporating H as an independent variable did not significantly improve the model's accuracy^[29]^, and some scholars have strongly opposed including H as an independent variable in allometric growth equations^[89]^. Instead, they favored using tree age or other variables, which also yielded satisfactory results. However, AGB equations using tree age as an independent variable were difficult to apply in natural forests, often leading to error propagation and greater uncertainty in estimates^[90]^, which, in turn, lowered the accuracy of AGB prediction models based on CW and H. These conclusions also explain why H was lower. In contrast, due to the mature DBH measurement technology, it remained stable in the field surveys, and the use of a model incorporating D would have higher accuracy. This indicated that the models using D are still the best choice in accessible areas.

Moreover, although increasing the number of independent variables would enhance the model fitting accuracy, it would also reduce the model's generalizability. Thus, when selecting independent variables, a balance must be struck between accuracy and generalizability to meet the requirements of various research scenarios. Therefore, selecting suitable independent variables for a study is critical, as an excessive focus on precision can be counterproductive. In practice, selecting easily measurable variables like DBH may better align with actual research needs^[28,91]^.

### How to use multi-source remote sensing data to improve accuracy

With the continuous advancement of close-range remote sensing and forest AGB estimation research, multi-source data fusion has emerged as a critical strategy for enhancing estimation accuracy. By leveraging the complementary strengths of different sensors, multi-source fusion effectively addresses the inherent limitations of single-source data, thereby substantially improving the precision and robustness of AGB estimation. Depending on the stage of integration and the data acquisition platform, fusion strategies are generally categorized into data-level fusion and feature-level fusion^[11]^. Data-level fusion involves the integration of raw data from different sensors or platforms prior to preprocessing. For example, ground-based and UAV-based point cloud datasets can be co-registered through spatial alignment techniques to create unified three-dimensional representations of forests^[92,93]^. This form of fusion was particularly effective at the single-tree scale, where it was widely employed to enhance structural parameter retrieval and AGB estimation^[80]^. Feature-level fusion, by contrast, entailed preprocessing data from multiple sources, extracting the relevant features, and then merging them into a composite dataset that synthesizes information across modalities. While spectral methods often struggle to capture key parameters for AGB estimation—such as H, DBH, and canopy metrics^[94,95]^—lidar provides direct, high-precision measurements of these attributes, along with detailed representations of the forest's vertical and horizontal structure^[61,96]^. Studies have consistently shown that integrating spectral and lidar data enhances estimation performance^[26]^. Feature-level fusion could be further subdivided into point-based and object-based approaches. Point-based fusion was typically implemented at the plot and stand scales, wherein lidar point cloud data were rasterized onto a standardized grid and overlaid with multi-spectral or hyperspectral imagery^[97]^. Object-based fusion, on the other hand, is more suited to the single-tree scale, involving the extraction of individual tree features and their subsequent integration across data sources. In addition, feature-level fusion could incorporate a wide range of auxiliary variables, including canopy closure, DEM, DTM, annual precipitation, mean annual temperature, and other non-vegetation factors such as climate conditions and soil fertility. The integration of these variables further strengthen the reliability and accuracy of estimating forest AGB^[98−100]^.

Future research on multi-source remote sensing data should focus on the following three areas. The first is the development of new methods to process and fuse data from different sensors and platforms via multi-source, cross-scale, and cross-platform fusion, aiming to improve the accuracy of AGB estimation. This includes addressing issues of data scale diversity and scale transformation, optimizing cross-platform fusion technologies, ensuring data quality, and reducing uncertainties. Second, we should focus on the application of artificial intelligence, particularly deep learning, in analyzing complex relationships within forest ecosystems. Deep learning methods have shown immense potential, especially in predicting forest AGB^[37,39]^. Future studies should explore the incorporation of cutting-edge deep learning algorithms from computer science into forest AGB estimations to meet AGB estimation needs across diverse regions and tree species. Lastly, there is significant variation among close-range remote sensing AGB estimation methods, and conclusions are often constrained by numerous factors. Therefore, determining the true values for verfying the accuracy remains a crucial issue. Harvesting methods have long been considered the most accurate and reliable, but in complex forest environments, ground-based field surveys are challenging and large-scale validation is not feasible. Currently, AGB estimation results based on close-range remote sensing techniques are primarily validated through plot sampling surveys or recent forest resource inventory data. These data are reported by provinces or regions, and due to varying levels of forestry development, there are considerable differences in accuracy and authenticity between the datasets.

## Conclusions

This meta-analysis systematically evaluated close-range remote sensing techniques for AGB estimation, and addressed uncertainties arising from variations in the spatial scale, forest type, and methodological approach. The findings indicated that ground lidar consistently achieved the highest estimation accuracy across study scales, while UAV lidar demonstrated superior suitability for stand-scale assessments. Sensor performance varied substantially across forest types, emphasizing the necessity of incorporating different forest types when constructing models. The accuracy of the three primary methodological categories (parametric methods, non-parametric methods, and allometric growth equation models) was broadly comparable. However, the inclusion of composite variables such as DBH and H in allometric growth equation models notably enhanced the explanatory power. This study highlighted the inherent limitations of single-source data and demonstrated that multi-source data fusion effectively mitigates signal saturation and error accumulation from fine to broad spatial scales, particularly in structurally complex forests. By integrating complementary sensor capabilities, the fusion method has been shown to establish a robust paradigm for high-precision estimations of carbon stocks. Looking forward, the advancement of close-range remote sensing systems should focus on three key directions: (1) establishing standardized protocols for cross-platform integration of point cloud and spectral data; (2) developing multi-scale inversion models that synergize three-dimensional structural features with deep learning algorithms; and (3) constructing a non-destructive accuracy evaluation framework based on terrestrial laser scanning validation. Collectively, our research will provide critical scientific support for the precise quantification of forest carbon sinks in pursuit of carbon neutrality goals.

## SUPPLEMENTARY DATA

Supplementary data to this article can be found online.

## Data Availability

All data underlying this study were systematically retrieved through a comprehensive meta-analysis of peer-reviewed literature (2010–2025). All data generated or analyzed during this study are included in this published article and its supplementary information files.
